# Liposome-Based Liquid Handling Platform Featuring Addition, Mixing, and Aliquoting of Femtoliter Volumes

**DOI:** 10.1371/journal.pone.0101820

**Published:** 2014-07-03

**Authors:** Hideaki Shiomi, Soichiro Tsuda, Hiroaki Suzuki, Tetsuya Yomo

**Affiliations:** 1 Graduate School of Information Science and Technology, Osaka University, Osaka, Japan; 2 School of Chemistry, University of Glasgow, Glasgow, United Kingdom; 3 ERATO, JST, Tokyo, Japan; 4 Faculty of Science and Engineering, Chuo University, Tokyo, Japan; 5 Graduate School of Frontier Biosciences, Osaka University, Osaka, Japan; Imperial College London, United Kingdom

## Abstract

This paper describes the utilization of giant unilamellar vesicles (GUVs) as a platform for handling chemical and biochemical reagents. GUVs with diameters of 5 to 10 µm and containing chemical/biochemical reagents together with inert polymers were fused with electric pulses (electrofusion). After reagent mixing, the fused GUVs spontaneously deformed to a budding shape, separating the mixed solution into sub-volumes. We utilized a microfluidic channel and optical tweezers to select GUVs of interest, bring them into contact, and fuse them together to mix and aliquot the reaction product. We also show that, by lowering the ambient temperature close to the phase transition temperature *T*_m_ of the lipid used, daughter GUVs completely detached (fission). This process performs all the liquid-handing features used in bench-top biochemistry using the GUV, which could be advantageous for the membrane-related biochemical assays.

## Introduction

With advances in micro/nano-scale sciences, a platform for liquid handling in a minute volume has been challenging.[1], [2] The fundamental liquid-handling procedures in biochemistry, such as adding, mixing, and aliquoting (dividing), have been achieved with volumes ranging from nano- to picoliters using droplet-based microfluidic systems.[3]–[5] The droplet-based microfluidic devices, which is often coined as digital microfluidics, have become established techniques for single-cell and single-enzyme experiments, where low-volume liquid handing and high throughput are necessary.[6]–[9] This trend of miniaturizing reaction containers further encouraged micro/nanotechnology researchers to develop microfluidics systems capable of handling femtoliter volume (typical diameter smaller than 10 µm). Reaction containers at this scale are utilized in nature by biological cells such as bacteria and eukaryotic subcellular organelles, and engineered femtoliter-scale liquid handling platforms are expected to facilitate not only improvements in the throughput and sensitivity of the above-mentioned technologies but also our understanding of how biochemistry occurs in such small spaces.

Several research groups have established techniques for producing femtoliter-scale water-in-oil droplets, which can be used as static reaction containers.[10]–[13] However, when one tries to achieve the liquid-handling procedures commonly used at the laboratory bench at this scale, it becomes extremely difficult. One major obstacle is the large interfacial tension of the oil-water boundary; as the size of the droplet becomes smaller, the droplet tends to maintain a spherical shape upon the application of shear force or other energy inputs. In contrast, living cells seem to readily use such tiny volumes by using the lipid bilayer as the compartment boundary. The plasma membrane of the cell can produce a pico- to femtoliter-scale compartment that can grow (increase its area), divide, and selectively allow molecules to permeate. Micro- to nano-scale vesicles are used in intracellular trafficking and signal transduction. This amazingly diverse utility of the lipid membrane as a dynamic compartment stems from its flexibility. With a typical bending modulus of 10^−19 ^J, a small energy input not much greater than the thermal fluctuation can deform and perturb the boundary, and yet the membrane is impermeable to many solutes. This feature has been utilized by several researchers to control the initiation of reactions in femtoliter to even attoliter volumes.[14]–[20] When the membrane of neighboring lipid vesicles in contact is perturbed, vesicles easily fuse together, and the internal contents mix and initiate the chemical reaction. Mixing is one important manipulation in reagent handling. However, aliquoting (dividing) the femtoliter volume has turned out to be a nontrivial task. The Orwar group has demonstrated that a giant lipid vesicle can be divided by physically dissecting the vesicle with a microfiber,[21] but this process could be highly laborious.

Previously, we reported that giant lipid vesicles, which contain inert macromolecules in their aqueous cores, spontaneously deform into a budded shape after electrofusion.[22] We proved that this deformation of the lipid membrane interface is caused by a purely physical phenomenon termed “the depletion volume effect”, in which the translational entropy of the encapsulated polymer is maximized. This system could be viewed as a dynamic artificial chemical and biochemical microreactor mimicking a living cell; encapsulated reagents are mixed together upon fusion, and the resultant products are divided and aliquoted upon the budding shape change of the membrane.

In this work, we capitalize on this physical phenomenon to engineer a femtoliter-scale liquid-handling platform. Use of lipid membrane could be especially advantageous for biochemical assays related to the membrane-bound proteins and other molecules.[23], [24] In this work in particular, we used a polymethylsiloxane (PDMS) microfluidic device and optical tweezers to selectively manipulate GUVs containing biochemical reagents. The macromolecules necessary for membrane deformation are essentially inert to various biochemical reactions, making this strategy useful in a wide range of applications. We demonstrate multiple rounds of chemical reactions with the proposed system to prove the feasibility of the present strategy.

## Materials and Methods

### Preparation of GUVs

We prepared various GUV populations containing different chemical and biochemical compounds via the water-in-oil (w/o) emulsion transfer method described previously.[22], [25] Briefly, we prepared water-in-oil emulsion with the aqueous solution of GUV as the water phase and with the liquid paraffin as the oil phase. The emulsion was layered on another water phase in the test tube, which was centrifuged to form GUVs. We chose two sets of compounds that fluoresce upon reagent mixing (Fig. S1) as the inner aqueous phases. In one case, we encapsulated an enzyme (β-galactosidase, 184 nM) in one population and a fluorogenic substrate (5-chloromethylfluorescein di-β-D-galactopyranoside, 100 µM; CMFDG, Invitrogen) in another population. Upon mixing, the enzyme hydrolyzes the substrate to produce fluorescein. In the second case, we encapsulated the fluorescent molecule calcein (10 µM) together with CoCl_2_ (200 µM) in one population and ethylenediaminetetraacetic acid (EDTA, 25 mM) in another population. In this case, the fluorescence of calcein in the first population is quenched by forming a complex with Co^2+^ ions. Upon mixing with EDTA, Co^2+^ is chelated, liberating the calcein molecule to emit green fluorescence. In all vesicle populations, an inert polymer (4 mM polyethylene glycol (PEG), 20 kDa molecular weight) was co-encapsulated. Glucose and sucrose with a total concentration of ∼500 mM were also enclosed, and the same concentration of glucose was present in the outer solution to adjust the specific density of the vesicle and for osmotic stability.[22] Furthermore, each vesicle population was marked by co-encapsulating near-yellow (R-phycoerythrin, 200 nM) and red (Transferrin Alexa Fluor 647, 3 µM, Invitrogen) fluorescent proteins for identification and visualization. The detailed compositions of the inner and outer solutions are described in Table S1.

### Experimental setup and procedure

**Figure 1:**
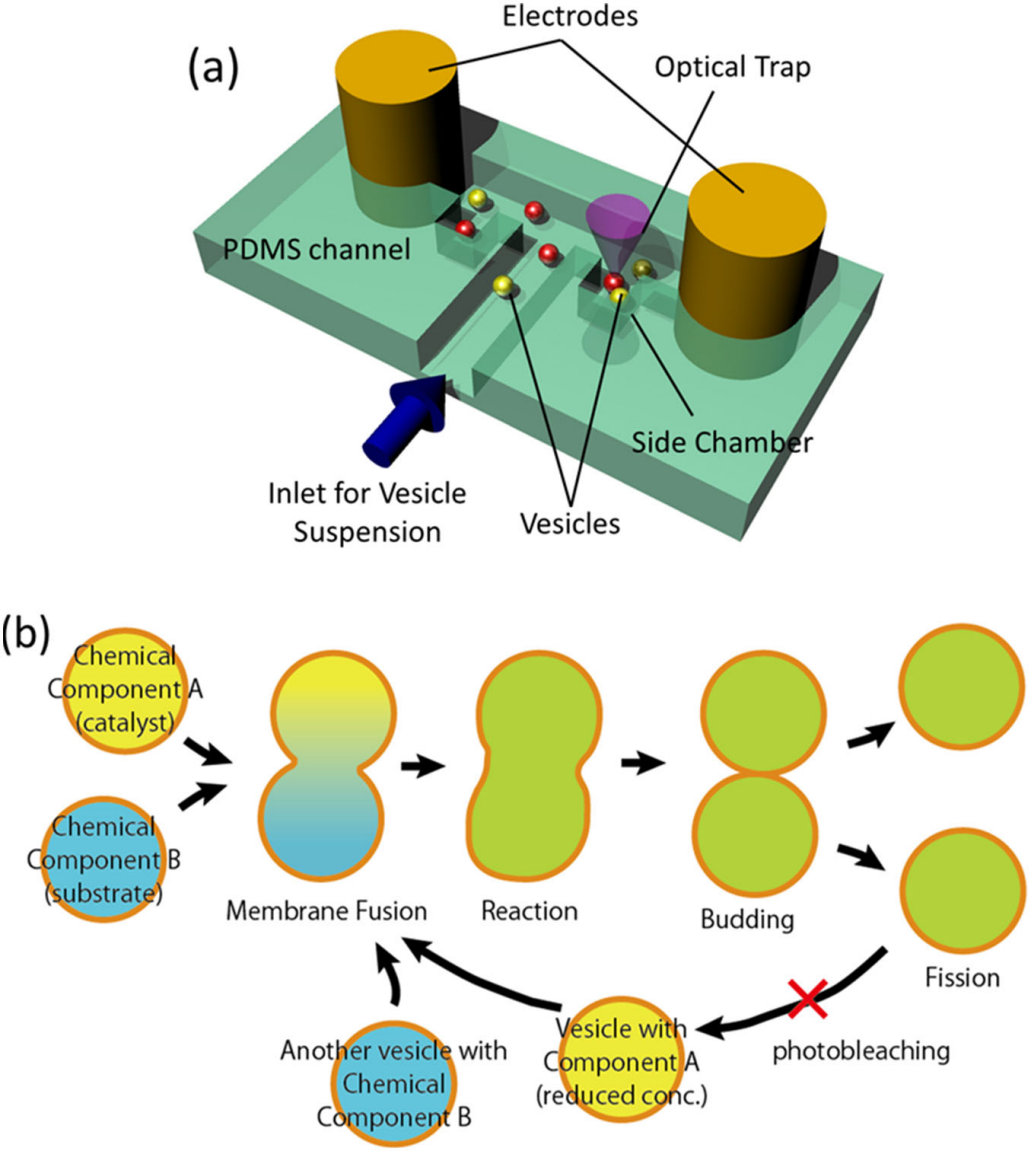
(a) Overview of the experimental setup. Giant vesicles are manipulated with optical tweezers and fused with an electrical pulse. (b) Schematic of the chemical-handling processes using GUVs. Reagent mixing is induced by fusion, and the reaction products are aliquoted by vesicle division. These processes are repeated for sequential (bio)chemical reactions. In the following experiments, reactions that fluoresce upon reagent mixing are used. The fluorescence in the GUV resulting from the first fusion and reaction is photobleached before the second reaction.

We fabricated a microfluidic channel system as shown in the schematic in Fig. 1a using a standard PDMS (Silpot 184, Toray Dow Corning) molding technique. After mounting the PDMS channel on the glass slide, two metal wires (0.75 mm diameter) used as electrodes were inserted into the two ends of the T-shaped part of the channel. The electrodes were connected to the signal generator for cell fusion (LF201, Neppa Gene). Manipulation and visual observation of GUVs occurred under a fluorescence optical microscope (either Nikon Ti or Olympus IX-71).

The two GUV suspensions prepared as described above were mixed and injected into a PDMS microfluidic channel. Two GUVs containing counterparts for a reaction and residing between electrodes were selectively moved to bring them into contact using optical tweezers with an infrared (1024-nm) laser (MMS-1064, Sigma Koki). An AC signal (150 V/cm, 1 MHz) was applied between the electrodes to ensure that the two vesicles were in close contact, as an attractive force is exerted due to dielectrophoresis. Next, short DC pulses (typically ∼6 kV/cm, 60 µs, 3 times) were applied to fuse the GUVs. After fusion, the reaction and the deformation of the fused GUV toward division was recorded using a CMOS digital camera (ORCA Flash 2.0, Hamamatsu Photonics). The fused vesicle was transferred into a side chamber using the laser tweezers to protect it from movement due to unexpected convection flow, especially during lengthy observation periods. For repetitive operation, new vesicles were brought from the inlet channel. We confirmed that those new vesicles were intact and did not experience the preceding electric pulses by observing the intensity of the encapsulated fluorescent maker, which remained at the same level before and after the application of the DC pulse. This consecutive manipulation can be repeated to sequentially continue the reaction (Fig. 1b). For testing conditions for the fission of daughter vesicles, a conventional hand-made chamber with copper-tape electrodes [22] was used.

## Results

### Enzyme reaction

**Figure 2:**
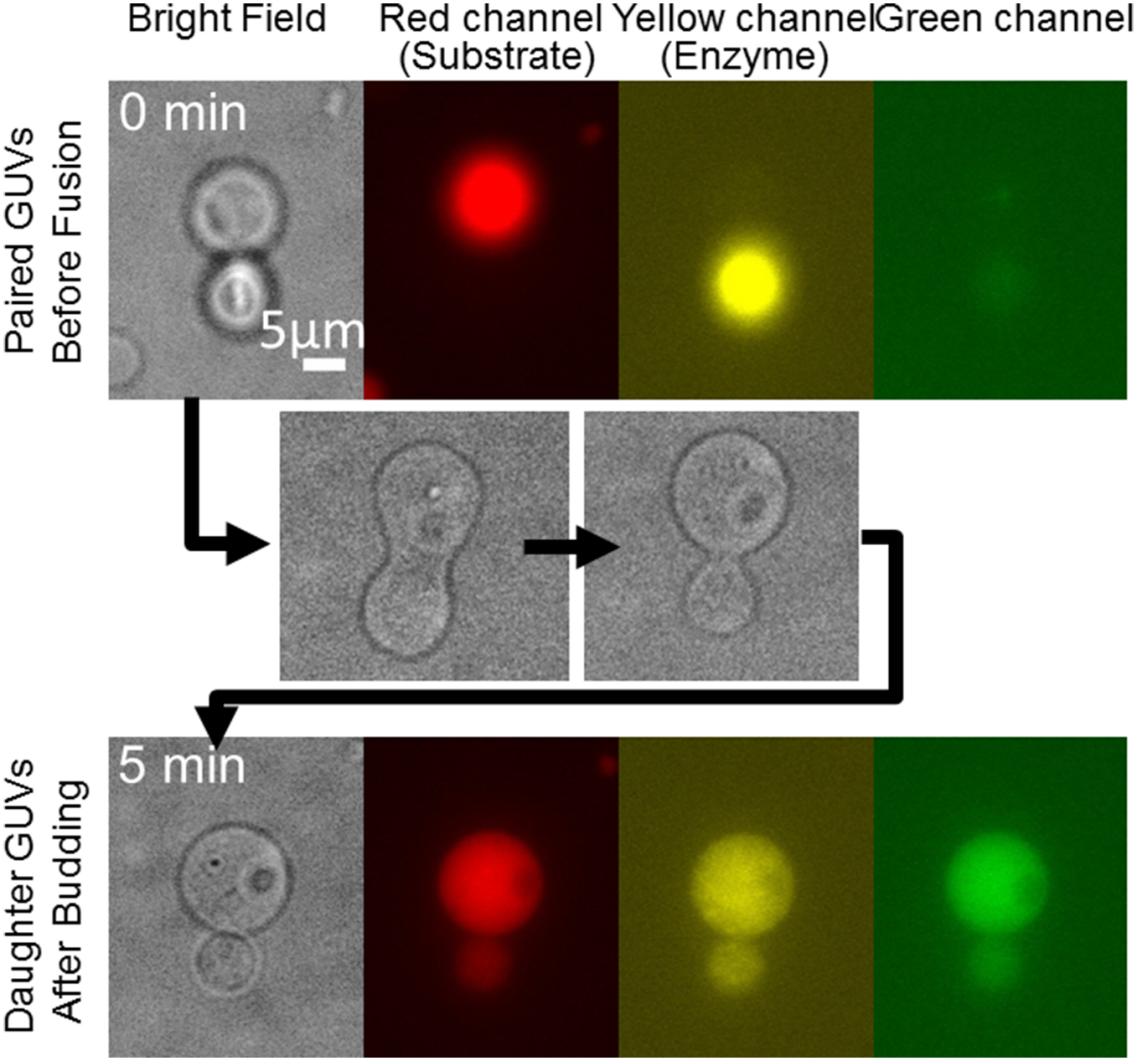
Fusion, reaction, and budding of vesicles. The upper images show GUVs before fusion. The red channel shows the marker for the substrate-containing GUV, whereas the yellow channel shows the marker for the enzyme-containing GUV. The middle images show the budding transformation process after electrofusion. The lower images show daughter GUVs after budding. The increased fluorescence in the green channel indicates the occurrence of the enzymatic reaction.

The upper micrographs in Fig. 2 show the brightfield and three different fluorescence images of GUVs brought into contact with the optical tweezers. In the red and yellow channels, fluorescence was emitted from one or the other of the two vesicles before fusion. Subsequently, the AC and short DC pulses were applied to induce vesicle fusion (the lower images in Fig. 2). The fused vesicle spontaneously transformed into a budded shape, with a peanut-like shape as an intermediate (middle images). After ∼5 min, the budding transformation completed, and a septum became visible between the two daughter vesicles. Under fluorescence observation, both fluorescent markers were present in both vesicles at lower concentrations. This observation demonstrates that the internal contents of the vesicles were mixed together. Another example of the same sequence of events, i.e., GUV fusion, internal content mixing, and budding transformation, is shown in Movie S1. After budding, the fluorescence intensity in the green channel from the hydrolyzed substrate was increased (Fig. 2), showing that the enzymatic reaction was triggered by mixing.

We previously reported the spontaneous vesicle deformation and demonstrated that it is due to an entropy-driven effect.[22] That is, as the curvature of the membrane increases (*i.e.*, as the membrane bends), the depletion volume near the membrane, which the inert macromolecule (20-kDa PEG in this experiment) cannot approach due to its size, decreases. Assuming that the inner volume of the GUV remains unchanged, this decrease of the depletion volume results in an increase of the volume containing the macromolecule. Because the chemical potential of the solution is lower than the solvent without solute, the total free energy of the system decreases as the membrane bends. This is equivalent to increasing the translational entropy of the macromolecules encapsulated in GUVs. If the free energy decrease due to entropy exceeds the energy necessary to bend the membrane, the GUV deforms to increase membrane curvature. In this work, we confirmed that the budding transformation also took place in the presence of the enzyme and its substrates for biochemical operation. The budding transformation never takes place without PEG being encapsulated [22].

### Complete fission

**Figure 3:**
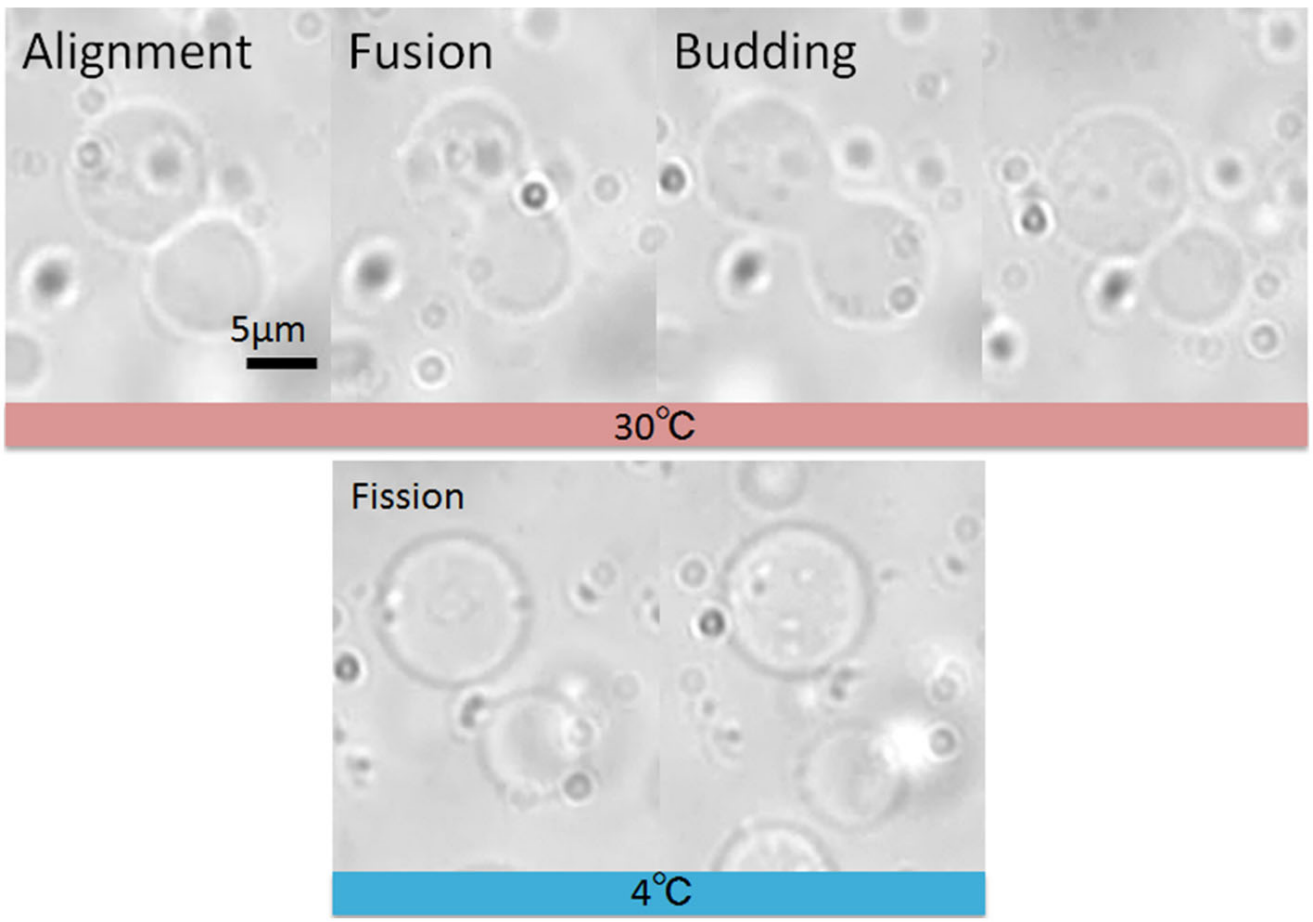
Brightfield micrographs showing the fusion, budding, and fission processes of GUVs. Fission (complete detachment) of daughter GUVs after budding was induced by lowering the temperature close to the *T_m_* of the lipids.

To repeatedly continue the procedures depicted in Fig. 1b, the two daughter vesicles generated by budding must be physically separated. In the previous study,[22] it was not clear whether the neighboring GUVs were completely separated, although diffusion of the fluorescently tagged proteins was not observed for at least 10 min. In the present study, we pulled these vesicles apart using optical tweezers and found that they were connected by a narrow lipid tube (See Movie S2). We postulated that the connection between the daughter vesicles stretches easily because the membrane is in the fluid state. Therefore, we placed the whole microfluidic device into a cold room (4°C) and microscopically observed vesicles after fusion. As shown in Fig. 3 and Movie S3, in experiments using a conventional electrofusion chamber [22], at this temperature the neighboring daughter vesicles drifted and separated from each other due to thermal fluctuations, typically a few minutes after the budding transformation. We found that, of 15 trials, approximately half of the budded vesicles (8/15) completely detached. It has been reported that lowering the temperature of membrane vesicles, from the liquid phase through the phase transition into the gel phase (*T* < *T*_m_), leads to the complete rupture and fission of the lipid neck, perhaps due to reduced line tension.[26], [27] Although the *T*_m_ of the main lipid used in this experiment (1-palmitoyl-2-oleoylphosphatidylcholine; POPC) was −2°C, we speculate the reported phenomenon occurred due to the added cholesterol, which is known to broaden the *T*_m_ of phospholipid membranes [28].

### Repeated Operation

**Figure 4:**
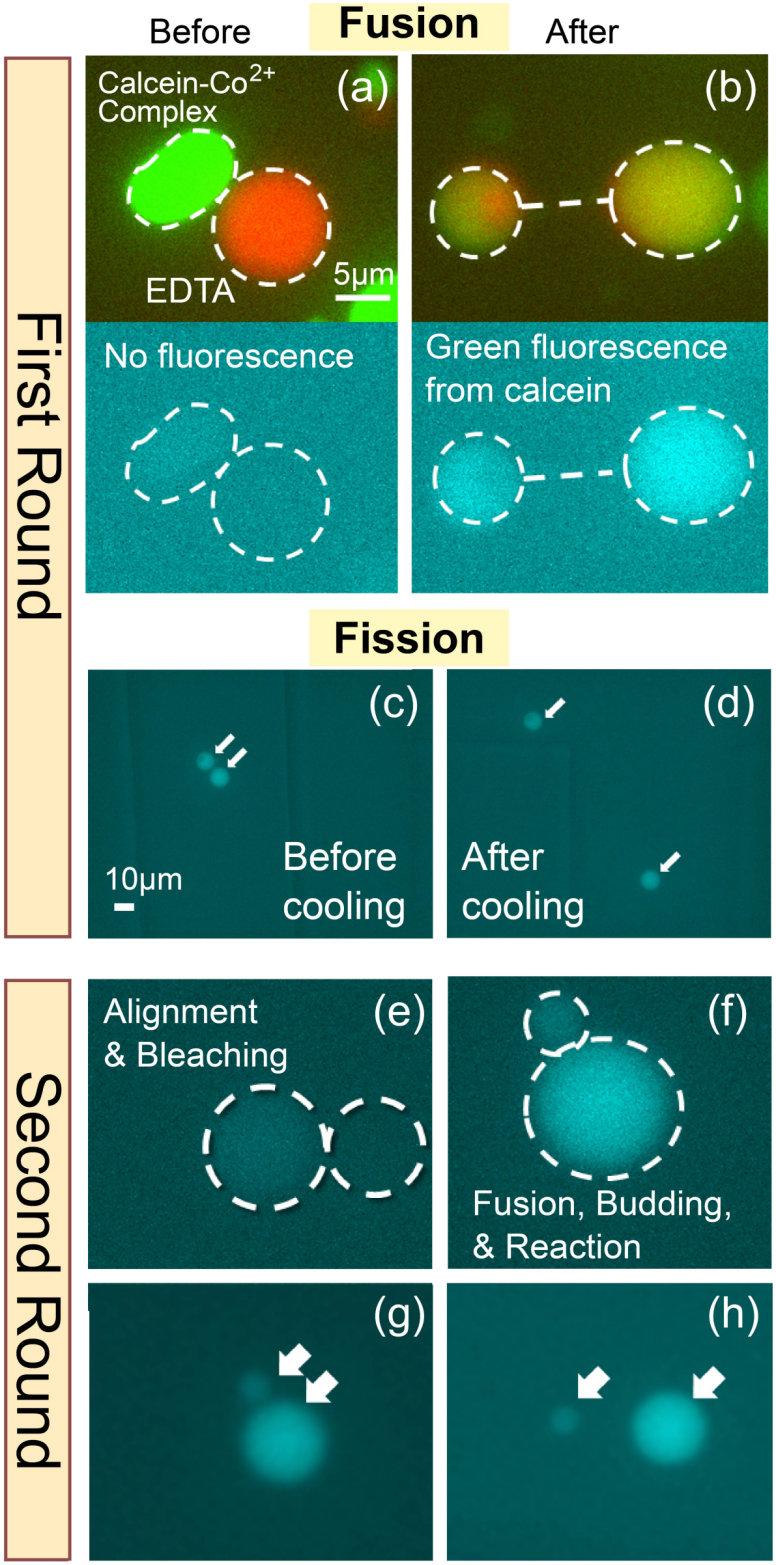
Fluorescence micrographs showing two successive rounds of vesicle fusion (mixing), reaction, and budding/fission (aliquoting). The 5-µm scale bar in panel (a) also applies to panel (b) and to panels (e)–(h), whereas the 10-µm scale bar in panel (c) also applies to panel (d).

Finally we demonstrated two sequential rounds of reagent mixing, reaction and aliquoting using the microfluidic and optical tweezers setup. Fluorescent micrographs from each step are shown in Fig. 4. The chelating reaction of the calcein-Co^2+^ complex, which exhibits green fluorescence upon mixing, was used for this experiment. In Fig. 4a, two GUVs, one containing the calcein-Co^2+^ complex and one containing EDTA, shown in the image in green and red, respectively, were aligned using the optical tweezers. At this point, no fluorescence was observed in the green channel, indicating that the fluorescence from calcein was quenched by the cobalt ion. After the application of electric pulses, the aqueous phases of the two vesicles fused together. Similar to the experiment in Fig. 2, the internal markers were observed in both daughter vesicles after the budding transition, and green fluorescence appeared as a result of the release of calcein (Fig. 4b). However, at room temperature, daughter vesicles were still connected by a lipid tube. The vesicle was then moved into a side chamber so that we could find the fused and budded vesicle later. We then placed the whole microfluidic device into the cold room. As a result, the connected vesicles had detached after ∼30 min (Fig. 4c–d). Next, the fluorescence of the liberated calcein was photobleached by applying UV light (Fig. 4e). Next, as the second round, another GUV containing EDTA was brought in contact with and fused to one of the daughter vesicles (Fig. 4f). The reappearance of green fluorescence demonstrated that the internal contents were successfully transferred in the second round of the operation. Finally, by again placing the device in the cold room, we induced the complete fission of the daughter vesicles.

## Discussion

We have successfully demonstrated, as a proof of concept, that use of GUVs as biochemical reaction containers enables the mixing and aliquoting of biochemical reagents at a femtoliter scale in a controlled manner. In fact, the fusion of vesicles is relatively easy and can be induced by various methods, as demonstrated elsewhere (fusogenic reagent,[15], [16] phase separation,[27] electrostatic interaction [14]). The most challenging task was to divide small aliquots of the reagent at the femtoliter scale. Although we demonstrated the feasibility of using the depletion volume effect, which can in principle be made inert to any reaction, we do not have precise control over the volumes of daughter vesicles after budding. In the elastic model of the lipid membrane, the elastic energy necessary to make a spherical vesicle has no scaling factor,[29] so that budded vesicles can have any size. Perhaps the addition of a scale-determining factor into the system would be a key advance for future development. Living cells seem to perform the mixing and aliquoting tasks easily when trafficking and transferring materials with the aid of membrane-bound proteins. We must thus learn and harness methods employed by nature to truly realize nano- to microscale biotechnologies.

## Supporting Information

Figure S1 **Schematics of the reporter reaction systems.** (a) The enzyme reaction, in which b-galactosidase hydrolyze the fluorogenic substrate upon vesicle fusion and internal content mixing. (b) The chelating reaction of Calcein-Co^2+^ complex.
(TIF)

Table S1 **Detailed components of the inner and outer solution of GUV populations.**
(XLSX)

Movie S1 **Fluorescent imaging of the fusion of two GUVs with diferrent dye.**
(MP4)

Movie S2 **Stretching of two adjuscent daughter vesicles after budding transformation.** Optical tweezers were used to pull GUVs. It turned out that they were connected by the tubular lipid structure.
(MP4)

Movie S3 **Complete fission of daughter GUVs which took place by lowering the ambient temperature to 4°C.**
(MP4)
