# Supplementary figures and images for: Liposome-Based Liquid Handling Platform Featuring Addition, Mixing, and Aliquoting of Femtoliter Volumes
Source: PLoS One. 2014 Jul 3;9(7):e101820. doi: 10.1371/journal.pone.0101820 (PMC4081812; doi:10.1371/journal.pone.0101820)

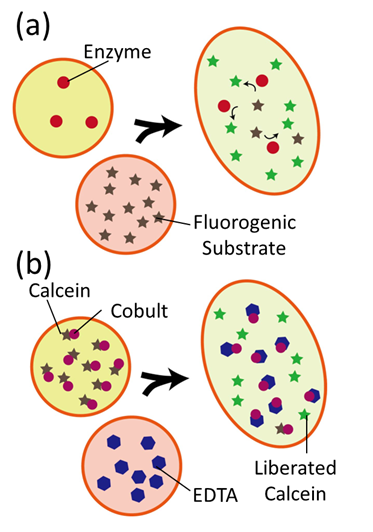

Supplement: Figure S1 — Schematics of the reporter reaction systems. (a) The enzyme reaction, in which b-galactosidase hydrolyze the fluorogenic substrate upon vesicle fusion and internal content mixing. (b) The chelating reaction of Calcein-Co2+ complex. (TIF) [file pone.0101820.s001.tif]
